# A systematic methodological review of non-randomised interventional studies of elective ventral hernia repair: clear definitions and a standardised minimum dataset are needed

**DOI:** 10.1007/s10029-019-01979-9

**Published:** 2019-05-31

**Authors:** S. G. Parker, S. Halligan, M. Erotocritou, C. P. J. Wood, R. W. Boulton, A. A. O. Plumb, A. C. J. Windsor, S. Mallett

**Affiliations:** 1 The Abdominal Wall Unit UCLH, GI Services Department, University College London Hospital, 235 Euston Road, London, NW1 2BU UK; 2 UCL Centre for Medical Imaging, 2nd Floor Charles Bell House, 43-45 Foley Street, London, W1W 7TS UK; 3 The Institute of Applied Health Research, College of Medical and Dental Sciences, University of Birmingham, Edgbaston, Birmingham, B15 2TT UK

**Keywords:** Ventral, Hernia, Methodology, Studies, Standardisation

## Abstract

**Background:**

Ventral hernias (VHs) often recur after surgical repair and subsequent attempts at repair are especially challenging. Rigorous research to reduce recurrence is required but such studies must be well-designed and report representative and comprehensive outcomes.

**Objective:**

We aimed to assesses methodological quality of non-randomised interventional studies of VH repair by systematic review.

**Methods:**

We searched the indexed literature for non-randomised studies of interventions for VH repair, January 1995 to December 2017 inclusive. Each prospective study was coupled with a corresponding retrospective study using pre-specified criteria to provide matched, comparable groups. We applied a bespoke methodological tool for hernia trials by combining relevant items from existing published tools. Study introduction and rationale, design, participant inclusion criteria, reported outcomes, and statistical methods were assessed.

**Results:**

Fifty studies (17,608 patients) were identified: 25 prospective and 25 retrospective. Overall, prospective studies scored marginally higher than retrospective studies for methodological quality, median score 17 (IQR: 14–18) versus 15 (IQR 12–18), respectively. For the sub-categories investigated, prospective studies achieved higher median scores for their, ‘introduction’, ‘study design’ and ‘participants’. Surprisingly, no study stated that a protocol had been written in advance. Only 18 (36%) studies defined a primary outcome, and only 2 studies (4%) described a power calculation. No study referenced a standardised definition for VH recurrence and detection methods for recurrence varied widely. Methodological quality did not improve with publication year or increasing journal impact factor.

**Conclusion:**

Currently, non-randomised interventional studies of VH repair are methodologically poor. Clear outcome definitions and a standardised minimum dataset are needed.

**Electronic supplementary material:**

The online version of this article (10.1007/s10029-019-01979-9) contains supplementary material, which is available to authorized users.

## Introduction

In the UK, 44,000 ventral hernia (VH) repairs were performed in 2010, increasing to nearly 50,000 in 2015, a 13% rise over 5 years [1]. With an ageing [2] and increasingly obese [3] population, the risk of incisional hernia post midline laparotomy has increased, from 8% in 1980 to 16% in 2012 [4]. Recurrence after a previous hernia repair is also high, with minimal improvement over the last 30 years [5]. VHs that repeatedly recur, have a wide ventral defect or are contaminated are known as complex VHs, and successful repair is extremely challenging [6].

This surge in prevalence and complexity of VH disease has attracted attention from academic surgeons and given rise to specialised university hernia centres [7]. As VHs are predominantly iatrogenic, it behoves surgeons to investigate both prevention and cure. This demands high quality research to generate robust and meaningful data. We have recently investigated the methodological quality of randomised controlled trials (RCTs) of VH repair [8] and found that studies frequently employed poor methods, risking bias. We discovered that studies collected highly variable data relating to the pre-, intra-, and post-operative variables and reported multiple poorly defined outcomes. In particular, there was no standardised definition for hernia recurrence, length of follow-up, or methods to diagnose recurrence. This current variation in reported perioperative variables and outcomes frustrates comparison of outcomes across different trials. These challenges would be greatly diminished if investigators adhered to a common set of reported variables and outcomes. Consequently, there is an urgent need to establish a standardised minimum dataset for trials of VH repair. Adopting such a dataset would facilitate data pooling and allow researchers to better explore the impact of patient demographics, hernia characteristics, and intra-operative variables on both operative and patient outcomes.

The fact that some surgical studies lack methodological rigour has been identified previously and a recent systematic review found that 62% of surgical journals do not require authors to adhere to recognised reporting guidelines [9]. Reporting tools have been designed specifically to enhance reporting of surgical interventions [10]. For this methodological review of non-randomised interventional studies in VH repair we designed our own methodological assessment tool for VH studies using a combination of reporting guideline tools already published (Downs and Black [11], ROBINS-I [12], Newcastle–Ottawa [12], TIDieR [10] and STROBE [13]) and our own expert knowledge of the VH literature.

The aim of this systematic review was to evaluate the methodological quality of non-randomised interventional studies of adults undergoing VH repair. We hypothesize that there is a lack of rigorous research in VH repair studies, as demonstrated in the aforementioned review of VH RCTs [8]. We further aim to establish evidence from non-randomised studies, that clear outcome definitions along with a standardised minimum dataset are required in this field of surgical science.

## Methods

### Registration and reporting

This systematic review is reported in line with the preferred reporting items for systematic reviews and meta-analyses (PRISMA) statement [14]. Ethical permission is not required by our centre for systematic reviews of available primary literature. A protocol was developed and registered with PROSPERO, the international prospective register of systematic reviews (CRD42016043071).

### Eligibility criteria

#### Study design

We included non-randomised interventional studies of VH repair. We anticipated finding fewer prospective than retrospective studies. To compare their methodological quality, we included all eligible prospective studies identified, matching each with a single retrospective study.

#### Participants

We included studies of adults. We excluded paediatric studies (defined as 18 years or less) since these are no representative of ‘typical’ VH patients. As our review was methodological, we included all hernia populations and included studies than restricted participants according to specific diseases, conditions, or metabolic disorders (e.g. a study of participants with BMI > 30).

#### Target condition

We defined VH as any anterior abdominal wall defect associated with abnormal protrusion of intra-abdominal viscera [15]. We, therefore, included a range from simple primary umbilical/epigastric to large complex hernias. Studies combining multiple types of hernia were eligible, as we were interested in how hernias were graded.

#### Interventions

All interventions addressing VH repair were eligible. So, we included all types of comparative study, including those comparing mesh, plane of mesh insertion, surgical technique, with/without component separation, with/without panniculectomy, etc. Studies comparing the same intervention with minimal alteration were also eligible (e.g. “double-crown” versus “single row” tacks for laparoscopic repair).

#### Comparators

All interventional comparators were eligible. Studies that compared an intervention to conservative management (i.e., non-operative management of VH) were excluded.

#### Outcomes

Any study outcome was eligible.

#### Timing

We stipulated no minimum follow-up.

#### Setting

All settings were eligible.

#### Language

We restricted our search to the English language.

### Information sources

We searched the PubMed database (US National Library of Medicine, National Institutes of Health, Bethesda MD, 20894, USA) from 1st January 2005 to 1st January 2018. Our prior experience of systematic review of clinical interventions suggests that this is the most comprehensive database and little additional benefit is gained from searching other databases.

### Search string

Our search string identified and combined the two following criteria:

1. To identify studies of VH disease including complex disease we used the MESH terms “hernia”, “abdominal hernia”, “umbilical hernia” and “ventral hernia”. These were combined with keywords: “abdominal wall reconstruction”; “herniorrhaphy”; “ventral defect” and “entero-cutaneous fistula”.
2. To identify studies of surgical techniques used for VH repair we used the MESH terms: “general surgery”; “reconstructive surgical procedures” and “surgical mesh”. This was combined with keywords: “pneumoperitoneum”, “botox”, “botulinium”, “two-stage”, “two step”, “staged repair”, “component separation”, “transversus abdominis”, “retro-rectus”, “bridging”, “bridge repair”, “silo”, “open” and “laparoscopic”.

Our complete search string is shown in Online Supplementary Material 1.

### Study records

#### Data management

Identified citations were entered into a spreadsheet (Microsoft Excel for Mac 2011 v. 14.5.9, Microsoft Corporation, Washington), and uploaded subsequently into a reference manager able to access online original articles directly (Mendeley Desktop v. 1.17, London, UK).

#### Citation management and screening

Citations were divided up into two equal groups. The first-half were screened by (SGP) and the second-half by (CPJW), both surgical fellows. They discarded articles that were “clearly unsuitable” (e.g. subject not VH), retaining any regarded as “uncertain” or “definitely possible”. These two latter groups were then combined and all assessed independently by SGP, CPJW, and RWB to identify all eligible studies. These were divided into methodological groups as follows: randomised controlled trials, non-randomised prospective interventional studies, non-randomised retrospective interventional studies. Any article where uncertainty persisted was discussed face-to-face with senior members of the research team (SH and SM). An exclusion log was kept at all stages.

**Fig. 1:**
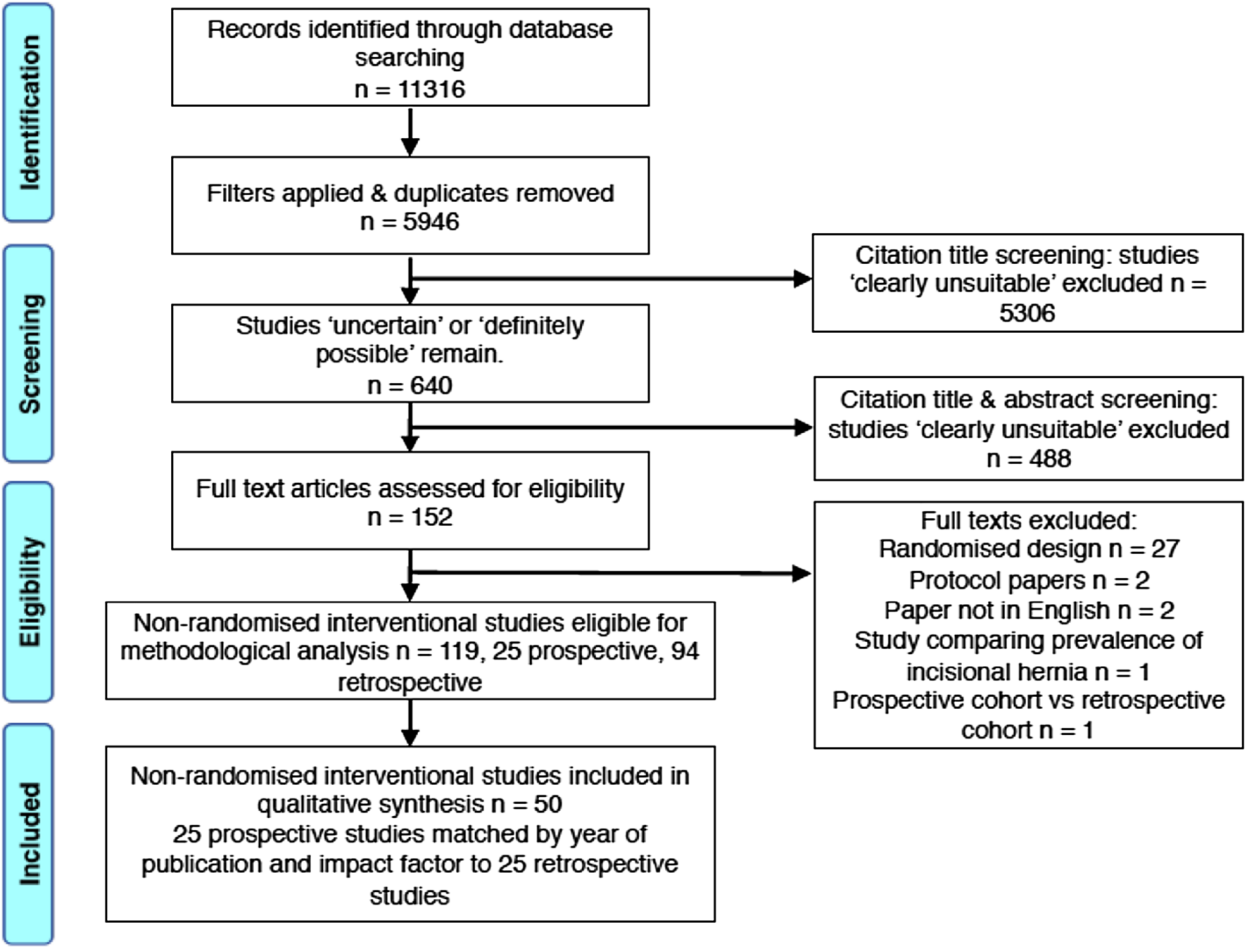
PRISMA diagram showing selection of non-randomised interventional studies for this review

The randomised controlled trials were excluded from the present review and reported elsewhere [8]. The following data were extracted from remaining studies; journal, impact factor, and publication year. Each prospective study was matched to a retrospective study. We attempted to match each prospective study to a retrospective study published in the same journal and year. If no studies met this criterion, we matched to retrospective studies published in the same journal but not in the same year. If no relevant articles were published in the same journal, we matched the prospective study to a retrospective study published in a journal with the closest impact factor. This procedure created a group of matched prospective and retrospective studies. A log of the matching process was kept. The flow of article selection is shown in the PRISMA diagram (Fig. 1).

#### Data extraction

SGP and ME extracted data independently from selected studies. To ensure consistency, data were cross-checked subsequently face-to-face and disagreement resolved by a third author, CPJW, and by senior authors, SH or SM, if discrepancy persisted. Data were entered into an Excel datasheet and categorised into broad groups as follows: introduction, study design, participants, reported outcomes, and statistical analysis.

### Data items

To assess methodological quality, we designed a methodological assessment tool relevant to our review by combining the most important data points from the following reporting and risk of bias guidelines tools: TIDieR [10], Downs and Black [11], ROBINS-I [12], STROBE [13], Newcastle–Ottawa [16]. Our tool is described in Online Supplementary Material 2. To analyse the introduction, we attempted to identify a rationale, primary aim or objective, and a pre-specified hypothesis with references to existing literature. To analyse design, we identified whether data were collected prospectively and according to a protocol. We also analysed whether studies described the equipment used and the proposed intervention adequately, using pre-specified criteria (Appendix 1 and 2, Online Supplementary Resource 2). We identified whether a primary outcome was described and whether a sample size calculation had been performed.

Regarding participants, we identified how patients were selected. We identified whether participants’ selection criteria or process was described adequately, and whether participants in intervention and comparator groups were drawn from the same population. To assess selection bias and to differentiate between patients meeting inclusion criteria versus number of participants included, we identified whether the study reported eligibility. We collected data on hernia morphology, assessing previous repairs were reported, maximal hernia width, defect area, whether primary or incisional hernias were reported, and whether a hernia grading scale was used. To assess participant characteristics, we identified whether a table of basic demographics was reported according to pre-specified criteria (Appendix 3, Online Supplementary Resource 2). To assess participant recruitment, we recorded whether recruitment start date, finish date, and end of follow-up date were reported. We identified whether the number of participants deviating from the intended intervention was reported.

Regarding reported outcomes, we assessed whether the assessor and/or participant were blinded to the intervention. Remaining information collected under this heading related to primary and secondary outcomes (see sections below).

For statistical analysis, we identified whether median length of follow-up and the number of participants with missing data were reported. We identified whether an adjusted analysis was performed and whether any adjustment factors were reported. We identified whether prediction estimates were reported for standard clinical variables. We also assessed whether confidence intervals were stated for all reported estimates. We identified whether an intention-to-treat or complete case analysis had been performed since this is most realistic in the clinical setting.

### Outcomes and prioritization

Our primary outcome of interest was hernia recurrence, so we extracted post-operative recurrence rates. We also extracted the timing of recurrence, definitions for VH recurrence, and the test method(s) used for diagnosis (for example, clinical examination, CT scan, and US scan). Our secondary outcomes were surgical site infection and surgical site occurrence, and we extracted definitions used to define them in the component studies. We also assessed whether a patient reported outcome measure was reported and, if so, its identity. Finally, manuscripts were reviewed to see whether a visual analogue scale (VAS) was used to assess post-operative pain.

### Risk of bias in individual studies

Existing reference tools were analysed [10–13, 16] and our assessment tool designed to identify the following categories of potential bias:

1. To assess selection bias we identified whether a study reported the number of eligible versus included participants.
2. To assess bias from intervention classification we included two questions from the TIDieR assessment tool [10]: (1) was a detailed description of equipment used reported (according to Appendix 1, Online Supplementary Resource 2)? And, (2) was a detailed description of the intervention reported (according to Appendix 2, Online Supplementary Resource 2)?
3. To assess bias regarding outcome measurement, we identified whether participants and/or assessor were blinded to the intervention.
4. To assess missing data bias, we identified if analysis was restricted to patients with full data.

Studies were assumed to be at low risk of bias if they adhered to all these criteria. ‘Unclear’ criteria were classified as moderate risk. ‘High’ risk of bias was determined by clear non-adherence to any criteria.

### Data synthesis

We used descriptive tables of frequencies for study items for prospective and retrospective studies. Box and whisker diagrams were used to present total methodological scores and to compare prospective and retrospective studies, enabling us to assess overall methodological quality. Scatter plots showed whether methodological quality was related to publication year and/or impact factor.

## Results

### Search results

Our initial search retrieved 11,316 results (Fig. 1). After applying filters (studies published 1st January 2005 to 1st January 2018; human; age > 18; English language), we excluded 5370 studies, leaving 5946. After title screening, 640 studies were categorised ‘definitely possible’ or ‘uncertain’, falling to 152 after abstract screening. After full text assessment, there were 119 non-randomised interventional studies; 25 prospective, 94 retrospective. Thus, after matching the prospective studies as described previously, the final review comprised 50 studies in total.

### Study demographics

Study demographics are shown in Table 1. The 50 studies reported 17,608 patients overall, 2800 (16%) prospective studies and 14,808 (84%) retrospective. Twenty-one studies (42% of total) were from the United States; 17 retrospective and 4 [17–20] prospective. Just five (10%) studies were multi-centre [21–25]. There were five categories of study with the same comparison groups: Nineteen laparoscopic versus open repair, five mesh versus suture repair [26–30], two primary fascial closure versus bridged repair [31, 32], two heavyweight versus lightweight mesh [33, 34], and two endoscopic component separation versus open component separation [35, 36]. Twenty-one (42%) studies (8 prospective, 13 retrospective) reported compliance with national or regional ethical standards. Three (6%) prospective studies [28, 37, 38] reported approval from an ethics committee, 3 more (6%) [18–20] referenced approval from the institutional review board, 1 (2%) study [39] reported ‘compliance with ethical standards’, and 1 (2%) study [40] reported compliance with ‘National Patient Rights Regulations’. Twelve (24%) of the retrospective studies reported approval from the institutional review board and 1 (2%) [29] reported approval from the hospital research ethics committee. Hernia type was specified by 32 (64%) studies; 18 prospective, 14 retrospective. Thirteen studies analysed both primary ventral and incisional hernia, eleven analysed incisional hernia, 3 analysed primary incisional hernia only [20, 38, 41], 3 analysed primary VH [27, 42, 43] and 2 analysed primary umbilical hernia only [28, 44].

**Table 1:** Demographics of the 50 non-randomised interventional studies included in the systematic review

Characteristic	Prospective study	No. of studies	Retrospective studies	No. of studies
Country of publication	USA [17–20], Spain [37, 45–47]	4	USA [22, 23, 25, 31, 32, 35, 48–58]	17
	Switzerland [59, 60], India [61, 62], Germany [33, 63], Belgium [34, 44]	2	Italy [41, 42, 64]	3
	Sweden [28], Italy [27], Poland [39], Norway [21], Singapore [65], Serbia [66], Austria [38], Turkey [40], Egypt [26]	1	France [24], UK [43], Germany [67], Pakistan [30], Saudi Arabia [29]	1
Multi vs single-centre	Multi centre [21]	1	Multi centre [22–25]	4
	Single centre [17–20, 26–28, 33, 34, 37, 39, 40, 44–47, 59–63, 65, 66]	24	Single centre [29–32, 35, 36, 41–43, 48–50, 52–58, 64, 67]	21
Study groups	Laparoscopic vs Open [17, 37, 40, 45, 46, 59, 60, 62, 63, 65]	10	Laparoscopic vs Open [25, 42, 43, 53, 55–57, 64, 67]	9
	Suture vs mesh [26–28]	3	Suture vs Mesh [29, 30]	2
	Heavyweight vs lightweight mesh [33, 34]	2	Primary fascial closure vs bridged [31, 32]	2
	Suture vs tack [18]	1	Endoscopic C/S vs Open C/S [35, 51]	2
	Sublay vs onlay [61]	1	Laparoscopic vs open C/S [22]	1
	Primary fascial closure vs bridged [38]	1	Panniculectomy vs no pannicculectomy [58]	1
	Bridging vs Primary fascial closure [IPOM vs IPOMplus] [39]	1	Posterior component separation vs anterior component separation [48]	1
	Autograft vs polypropylene mesh [66]	1	Polyester mesh vs PTFE [49]	1
	Single incision vs standard laparoscopic [21]	1	Concomitant vs no concomitant procedure [23]	1
	Flex HD vs Alloderm [19]	1	Mesh vs mesh + pedicle flap [41]	1
	Barbed suture and mesh vs mesh [20]	1	Suture vs tack [50]	1
	Fibrin sealant vs no fibrin sealant [47]	1	Permacol vs alloderm mesh [52]	1
	Open ventralex patch vs sublay mesh [44]	1	Ventralight ST vs control group [24]	1
			Laparoscopic vs robotic [54]	1
Hernia type	Primary ventral hernia [27]	1	Primary ventral hernia [42, 43]	2
	Primary umbilical hernia [28, 44]	2	Primary incisional hernia [41]	1
	Primary incisional hernia [20, 38]	2	Incisional hernia [23, 29, 49, 55, 67]	5
	Incisional hernia [19, 34, 59, 60, 62, 66]	6	Primary and incisional hernia [24, 30, 50, 53, 57, 64]	6
	Primary and incisional hernia [39, 40, 45–47, 63, 65]	7	Unclear [22, 25, 31, 32, 35, 36, 48, 52, 54, 56, 58]	11
	Unclear [17, 18, 26, 33, 45, 61]	7		

### Risk of bias assessment

**Fig. 2:**
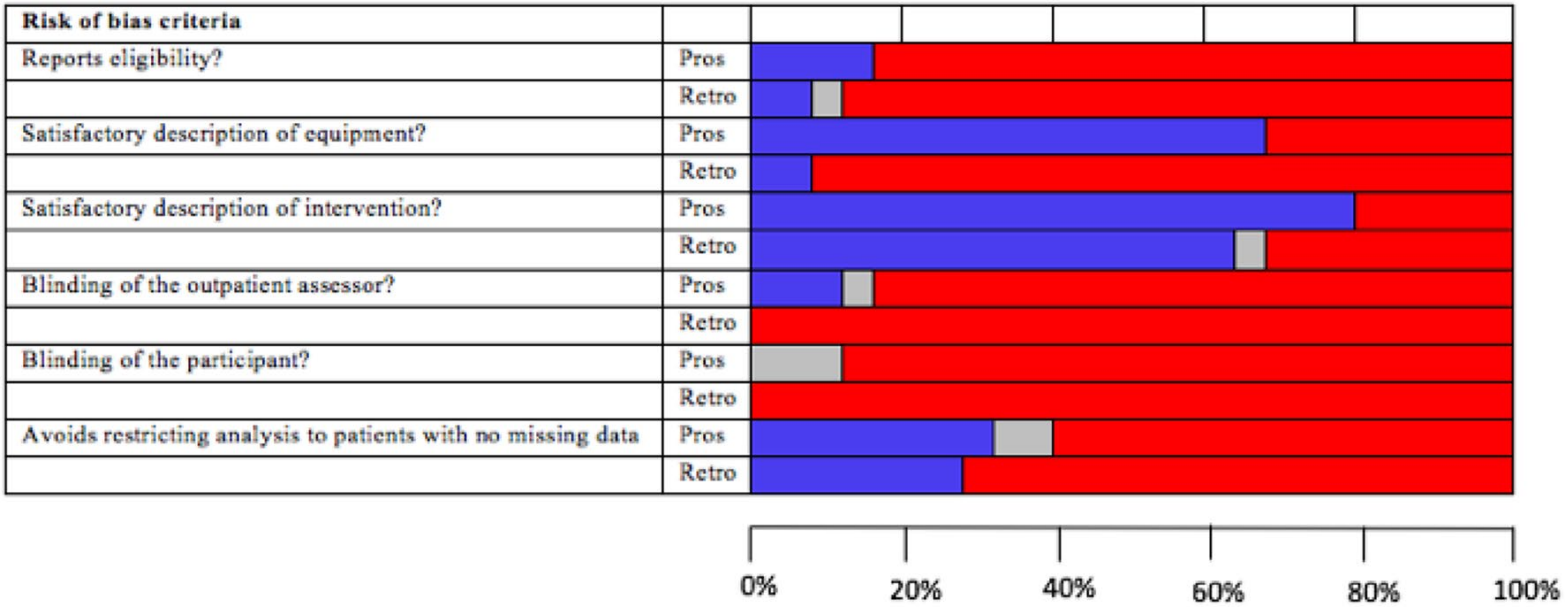
Graph of risk of bias item for prospective and retrospective studies (blue—studies reporting the criteria, grey—unclear, red—studies omitting the criteria)

All studies were rated as at high risk of bias. Figure 2 shows that this was mostly due to unblinding of both participants and assessors; only three (6%) studies [19, 47, 60], all prospective, achieved blinding for both these criteria. Although we aimed to assess selection bias, only six studies reported patient eligibility; four prospective [38–40, 60], two retrospective [29, 48].

### Methodology scores

Online supplementary resource 3 shows tabulated results from data extracted.

**Fig. 3:**
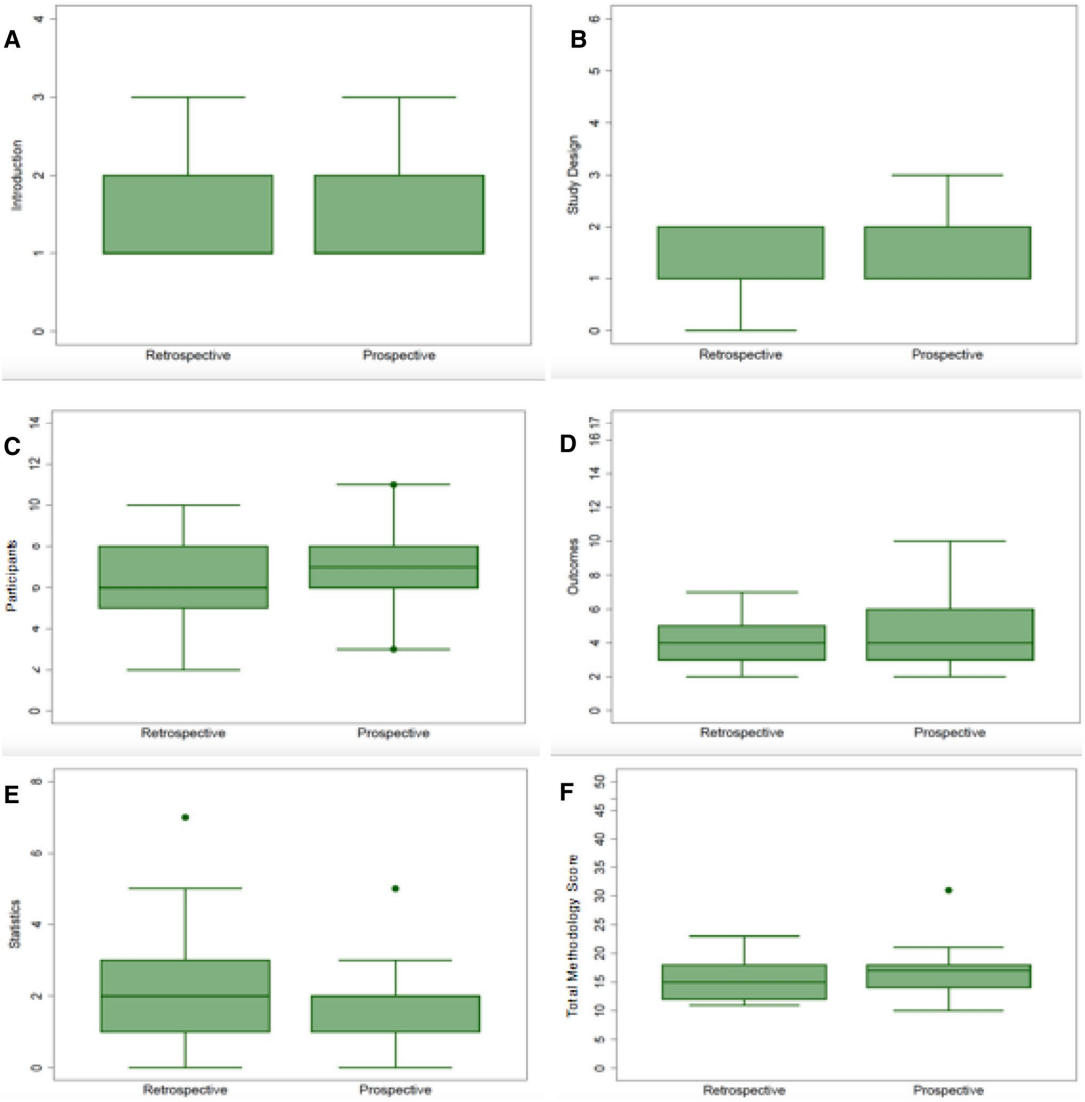
Box and whisker plots showing methodology scores for prospective and retrospective studies. **a** Introduction, **b** study design score, **c** participants score, **d** outcomes score, **e** statistics score, **f** total methodology score

**Fig. 4:**
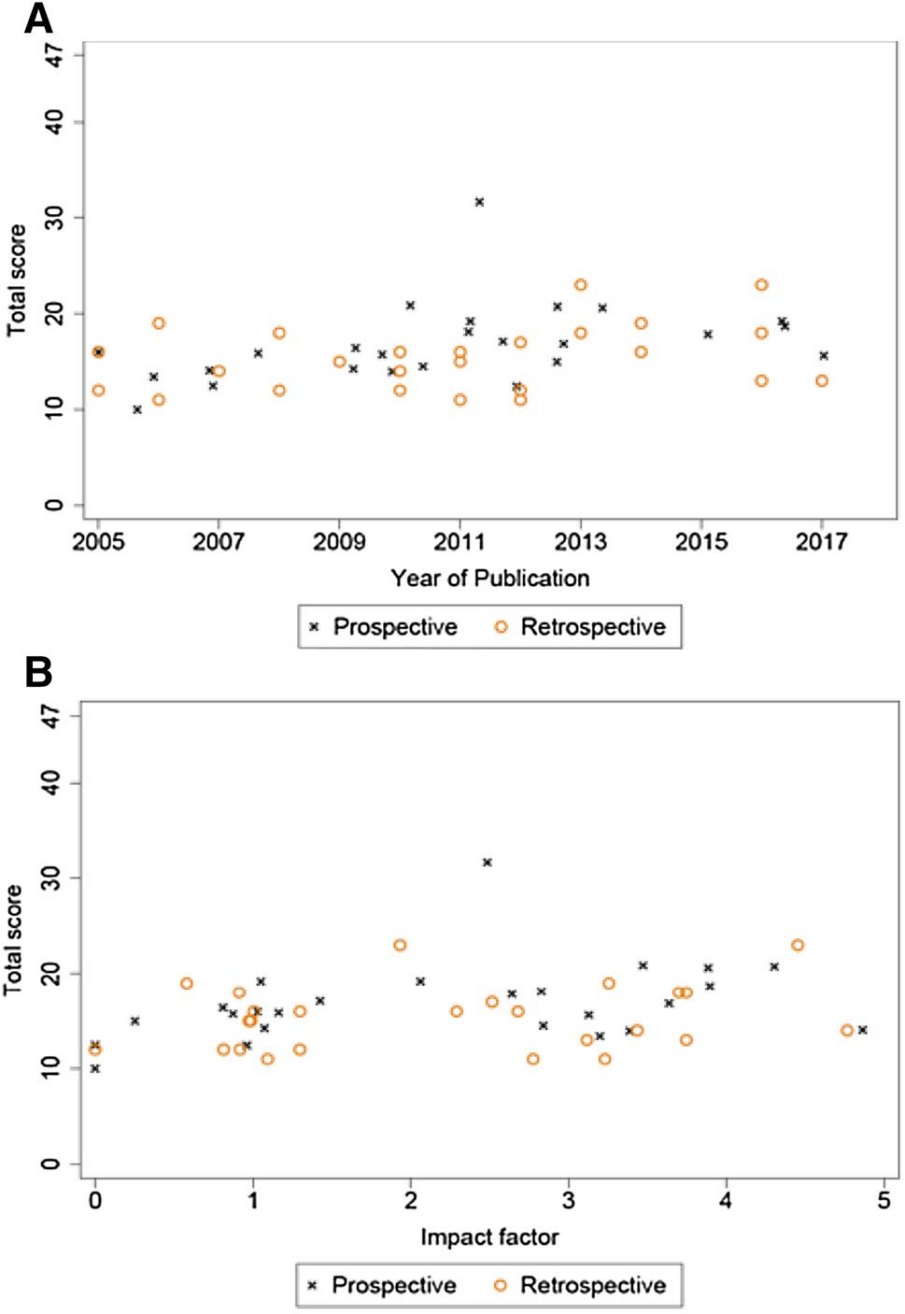
Scatter plots comparing methodological scores for prospective and retrospective studies. **a** Impact factor versus total methodology score, **b** year of publication versus total methodology score

As our data extraction sheet had 46 items, the maximum possible methodology score for any single study was 46. Total and sub-category median methodology scores with their interquartile ranges (IQRs) are depicted using Box plots in Fig. 3. The overall median score was 16 (IQR: 14 to 18), with a range of 11 to 31. Prospective and retrospective studies had median and IQRs of 17 (IQR: 14–18) and 15 (IQR: 12–18), respectively, with prospective studies having marginally better average methodological quality. For the sub-groups ‘introduction’, ‘study design’ and ‘participants’ prospective studies achieved higher median scores relative to the matched retrospective studies with median scores of 2 vs 1, 2 vs 1, 7 vs 6, respectively. For the subgroup ‘reported outcomes’ prospective and retrospective studies had equal median scores, 4 vs 4. In the ‘statistics’ subgroup the retrospective and prospective median scores were 2 vs 1 (Fig. 3). Scatter plots of methodological quality against publication year and impact factor (Fig. 4) showed no clear relationship for either prospective or retrospective studies. One study, Kurmann et al. [60], scored 31 and was 8 points higher than the next best methodological score.

## Introduction

All 50 studies (100%) provided a scientific rationale for their purpose. Twenty-nine studies (58%) described a primary aim or objective, with improved reporting for prospective (18 studies, 72%) versus retrospective (11 studies, 44%) studies. Only 3 studies [17, 32, 48] provided a hypothesis, and none of these referenced their hypothesis to the literature.

### Study design

No study (0%) stated that a study protocol had been published or written. Studies were generally poor at accurately describing the equipment used for hernia repair but were informative about the interventions performed. Nineteen (38%) and 36 (72%) studies reported these criteria, respectively. Only 18 (36%) studies defined a primary outcome, with similar proportions for prospective and retrospective studies; 8 (32%) vs 10 (40%). Only 2 (4%) studies performed a power calculation [38, 47].

### Participants

Thirty-five (70%) studies reported selection criteria beyond elective VH repair, time and place. Only 17 (34%) studies reported a basic list of baseline characteristics meeting our pre-specified criteria (Appendix 3, Online Supplementary Resource 2). Amongst the 34 (68%) studies that did report baseline characteristics (including the 17 studies that met our criteria), 18 (36%) studies showed equivalence between the intervention and comparator groups, whereas 16 (32%) studies reported a difference in one or more baseline characteristics indicating a difference in the group populations. In 16 (32%) studies no comparative analysis of baseline characteristics was performed.

Reported hernia characteristics also varied. Excluding studies that included only primary hernias (8 studies, 16%), the number of prior hernia repairs was only reported in 18 out of 42 (43%) studies. Twenty (40%) studies reported maximal hernia diameter, 12 (48%) prospective and 8 (32%) retrospective. Hernia defect area was reported by 21 studies, again with no detectable difference between the prospective and retrospective studies; 9 (36%) vs. 12 (48%). Thirty-two (64%) studies stated whether hernias were primary, incisional, or both, leaving 18 (36%) that did not state the hernia type included. Only 3 studies [24, 46, 60], graded hernias using either the EHS scale [24, 60] or their own pre-specified scale [46].

Participant recruitment start and finish dates were reasonably reported with 36 (72%) studies reporting both. In contrast, no study reported the end of follow-up date and only 18 (36%) reported the number of deviations from the intended intervention.

### Reported outcomes

Hernia recurrence rate was reported in 47 (94%) studies. Three retrospective studies [23, 54, 55] did not report recurrence. However, only 9 (18%) studies defined recurrence; 4 (16%) prospective and 5 (20%) retrospective. None of these studies used the same definition and none referenced a definition of recurrence (Table 2). Two studies [26, 59] reported recurrence but the overall follow-up duration was unclear. Of the remaining 45 studies, recurrence rate, follow-up duration, and detection method varied. Follow-up duration ranged from 3 [47] to 81 months [28], with a median of 27 months. Ten (20%) studies reported a follow-up of between 6 and 12 months. Follow-up duration for the remaining 35 (70%) studies lacked any consistency (Online Supplementary Resource 3). In 21 (42%) studies the follow-up duration differed between treatment arms. Fifteen different methods to detect recurrence were reported across 37 (74%) studies (Online Supplementary Resource 3), ranging from re-operation rate [33] to telephone interview [64]. Seven different detection methods were reported by prospective studies versus 12 different methods for retrospective studies. The most prevalent method used to detect recurrence was clinical assessment followed by a CT scanning if a recurrence was suspected.

**Table 2:** Nine definitions of hernia recurrence encountered in the systematic review

Prospective studies	Hernia recurrence definition	Referenced?
Kurmann et al. [60]	‘Recurrence was defined as any abdominal wall gap with or without bulge that is not covered by mesh in the area of the postoperative scar’	No
Anadol et al. [40]	‘Recurrence was defined as the presence of a defect and/or lump in the original location’	No
Moreno-Egea et al. [37]	‘Hernia recurrence was defined on physical examination and confirmed on CT’	No
Bochicchio et al. [19]	‘We defined a true hernia recurrence as herniation of bowel or omentum through a defect in the biological mesh or through a defect at the mesh/fascial interface after the initial operation’	No

Retrospective studies	Hernia recurrence definition	Referenced?
Al-Salamah et al. [29]	‘Recurrence was defined as any fascial defect, palable or detected on CT scan and located within 7 cm of the site of hernia repair’	No
Jin et al. [31]	‘Patients with recurrent hernias were defined as requiring another hernia reoperation or noting a significant bulge’	No
Ballem et al. [57]	‘recurrence was defined by the presence of a new or similar bulge which increased in size upon straining’	No
Booth et al. [32]	‘Recurrent hernia was a contour abnormality associated with a fascial defect’	No
Iacco et al. [52]	‘Recurrence was defined by the presence of a bulge on physical examination, imaging, or by patient self-reporting’	No

Surgical site infection (SSI) was reported by 32 (64%) studies. However, only six (12%) studies, three prospective [19, 28, 60] and three retrospective [29, 53, 57], defined SSI with only three definitions referencing the literature [19, 57, 60]. Two definitions used Center for Disease Control (CDC) wound infection criteria [19, 60], one study referenced NSQIP criteria [57], and the remaining three unreferenced definitions differed [28, 29, 51]. Surgical site infection was reported using an anecdotal grading scale by one study [58]. While one study provided the CDC SSI definition but the results then failed to use this for reporting wound infection rates [19].

Surgical site occurrence (SSO) was reported by four (8%) studies [35, 36, 40, 54]. Only one study [36] defined SSO but without providing a reference. Ten (20%) studies, seven prospective and three retrospective [24, 30, 50], stated patient reported outcomes. Two used the EQ-5D questionnaire [34, 44], one used the French Hernia Club questionnaire [24] and the remaining seven asked ad hoc outcome questions (e.g. time to normal activity, time to return to work). Nine (18%) studies used visual analogue scores to assess pain.

### Statistics

Forty-five (90%) studies reported follow-up duration. Multivariable adjusted analysis for hernia recurrence was reported by 10 studies; 7 retrospective and 3 [17, 18, 60] prospective. All 3 prospective studies [17, 18, 60] reported the adjustment factors compared to 5 of 7 for retrospective studies [23, 32, 36, 52, 53]. Eight (16%) studies reported confidence intervals for odds ratios and hazard ratios; 6 [24%] retrospective and 2 (8%) prospective [17, 18]. Only one study [61] reported a complete-case analysis with 100% follow-up at 24 months. No study used imputation to handle missing data so analysis was limited to patients with complete data.

## Discussion

In our first methodological systematic review [8], we found that reported variables in randomised controlled trials (RCTs) of VH were heterogenous and lacked standardisation, concluding that clear outcome definitions and a standardised minimum dataset are needed if VH research is to be clinically useful and methodologically credible. Because RCTs are the highest level of evidence [68], we can hypothesise that perioperative variables reported in non-randomised interventional studies of VH repair would be at least as deficient. Therefore, for the present review our emphasis was firmly upon assessment of study methodology. To achieve this, we designed a specific methodological assessment tool using published guidelines [10–13] (Online Supplementary Material 2).

We found that there was no generally accepted definition of hernia recurrence, no standardised test methods to detect recurrence, no standardised length of follow-up, no universally accepted definition for both surgical site infection (SSI) or surgical site occurrence (SSO), and no standardised evaluation tools for post-operative quality of life and pain. General markers of poor methods included an absence of study protocols and power calculations. This lack of standardisation and methodological vigour limits the validity of published results and, furthermore, impacts upon meta-analytical synthesis.

Perhaps the most pressing issue is a lack of definitions for study outcomes. Historically, the most studied outcomes are surgical site infection (SSI), surgical site occurrence (SSO), and hernia recurrence [69], yet we found researchers defined these items poorly. Regarding hernia recurrence, only 9 (18%) studies defined this and none of these used a standardised definition or referenced the literature. Similarly, methods to detect recurrence and follow-up duration varied. This lack of consensus regarding assessment timing, definitions for recurrence, and test methods used limits the utility of study findings. We advocate using the EHS definition for recurrence [15], *‘a protrusion of the contents of the abdominal cavity or pre*-*peritoneal fat through a defect in the abdominal wall at the site of a previous repair of an abdominal wall hernia’* as a broad definition for recurrence. However, it is imprecise and an additional definition of recurrence for VH trials that is far more precise and stipulates the exact findings on physical examination and includes the use of imaging to increase accuracy requires development [70]. Indeed, our previous review found that studies employing cross-sectional imaging reported double the hernia recurrence rate than other studies [8]. This supports urgent requirement for standardised detection methods in addition to definitions.

Similarly, we found that SSI and SSO were seldom defined and, even then, rarely referenced standardised definitions form the literature. These findings will not surprise hernia academics since they echo a recent review by Haskins et al. [71], who stated that of the 50 most cited papers describing VH repair, only 9 (18%) used standardised definitions for SSIs and SSOs. Haskins went onto propose definitions for SSI, SSO and SSOPI (surgical site occurrence requiring procedural intervention) that should be adopted by all studies of VH repair. The response from DeBord et al. [72] stated difficulties with the proposal but accepted the need for a “common language”. This editorial concluded by calling for an ‘international task force’ to establish common language for reporting wound complications in the field of abdominal wall reconstruction. We support this.

As well as identifying a paucity for defining outcomes, our methodology review identified additional major reporting deficiencies. No study mentioned writing a protocol, only 2 (4%) performed a power calculation, and only 18 described a primary outcome. These factors are pivotal to good-quality research. Protocols ensure that research is pre-planned and not haphazard, are important for research governance, and demonstrate that authors recognise that ‘quality control needs to be built in from the start rather than the failures being discarded’ at the end [73]. Power calculations are essential; small samples risk type 2 errors whereas too large a sample results in unnecessarily large and costly research, wasting time and effort. Just 18 studies described a primary outcome, an item fundamental to reporting research. In essence, non-randomised interventional studies of VH repair need improved study design and reporting to produce meaningful results.

Surgeons performing such studies should make concerted efforts to reduce bias. We deemed all 50 studies included in this review at high risk of bias. For example, good research practice demands eligibility criteria and keeping a screening log. However, only six studies reported eligibility and when they did so it was implied rather than reported specifically (e.g. ‘57 patients were diagnosed with incisional hernia, 44 underwent surgical repair’ [59]), leaving exclusion criteria in doubt. Poor reporting of ‘eligibility’ exposes studies to concern about potential for selection bias. In general, prospective studies described both the equipment and the intended intervention well and, as a consequence, were at low risk of bias regarding classification of interventions. In contrast, retrospective studies described interventions poorly, suggesting high risk of bias in this category. Retrospective studies cannot control the exact equipment and intervention that was performed on each participant. Studies scored poorly for blinding participant and assessor. While blinding of surgical studies can be difficult, visible skin changes give no clue as to where a mesh was placed or its nature or whether a component separation was performed. Accordingly, blinding should be possible for many hernia studies.

We found that recent publication or higher journal impact factor did not improve quality. This is disappointing because STROBE [13], Newcastle–Ottawa [16], and TIDieR [10] guidelines were published over the time-span of our review, suggesting that hernia researchers are unaware of these recommendations and not party to efforts to improve research quality over the last 20 years [74]. The Ventral Hernia Working Group’s classification of SSO was published in 2010 [69], which we would expect hernia researchers to endorse and use. Systematic reviews of other specialties have demonstrated improved methodology [75] and scoping reviews have shown quality improvement throughout the profession with both publication date and impact factor [76]. As VHs become increasingly prevalent [6], combined with high recurrence rates, these results highlight an urgent need to improve methodology in non-randomised interventional studies of VH repair.

This systematic review has identified a need to construct a standardised minimum dataset for non-randomised VH trials (which greatly outnumber randomised trials). Definition of core variables and outcomes is vital to move the academic hernia community forwards. This endeavour will require international collaboration across academic hernia surgeons. Once achieved, such a minimum dataset will enable trials and registries to report the same peri-operative variables and outcomes, which will facilitate comparisons across them via meta-analysis and multivariate logistic regression, improving our understanding of how each perioperative variable effects outcome. In research generally, there is a worldwide move towards establishing minimum datasets [77, 78]. In this review, and our review of randomised trials [8], we have established evidence that the data collected is currently highly heterogeneous and undefined; clear outcome definitions and a standardised minimum dataset are warranted.

## Conclusion

This systematic review is the first methodological review of non-randomised interventional VH studies. The results show that there is a lack of methodological rigour of both prospective and retrospective VH studies. In addition, methodological quality did not improve with publication year or journal impact factor. Studies failed to write protocols prior to implementation, a power calculation was seldom performed, and there was a general lack in defining a primary outcome. Furthermore, studies defined hernia recurrence, surgical site infection and surgical site occurrence poorly and used variable detection methods and grading scales. To solve this, a standardised minimum dataset with a standardised set of peri-operative variables, defined methodology and standardised outcome definitions are needed.

## Electronic supplementary material

Below is the link to the electronic supplementary material.

Supplementary material 1 (PDF 106 kb)

Supplementary material 2 (PDF 406 kb)

Supplementary material 3 (PDF 392 kb)
