# Supplementary material for: A systematic methodological review of non-randomised interventional studies of elective ventral hernia repair: clear definitions and a standardised minimum dataset are needed
Source: Hernia. 2019 May 31;23(5):859–72. doi: 10.1007/s10029-019-01979-9 (PMC6838456; doi:10.1007/s10029-019-01979-9)
Supplement: Supplementary file 1 — Supplementary material 1 (PDF 106 kb) [file 10029_2019_1979_MOESM1_ESM.pdf]

Online supplementary resource 1

A systematic methodological review of non-randomised interventional studies of elective ventral hernia repair: Clear definitions and standardised datasets are needed

Parker S.G[1], Halligan S[2], Erotocritou M[1], Wood C P J[1], Boulton R.W[1], Plumb A A O[2], Windsor A C J[1], Mallett S[3].

1. The Abdominal Wall Unit UCLH, GI Services Department, University College London Hospital, 235 Euston Road, London, NW1 2BU.
2. UCL Centre for Medical Imaging, 2nd Floor Charles Bell House, 43-45 Foley Street, W1W 7TS.
3. The Institute of Applied Health Research, College of Medical and Dental Sciences, University of Birmingham, Edgbaston, Birmingham, B15 2TT.

Corresponding Author: Mr Samuel G. Parker
Email: samgparker@nhs.net, Mobile: 07814136705 ORCID: 0000-0002-3710-9953

Search strategy:

Our complete search string:

((((((((("General Surgery"[MESH]) OR "Reconstructive Surgical Procedures"[MESH])) OR ((("pneumoperitoneum"[Title/Abstract]) OR "botox"[Title/Abstract]) OR "botulinum"[Title/Abstract])) OR (((("two stage"[Title/Abstract]) OR "stage repair"[Title/Abstract]) OR "staged repair"[Title/Abstract]) OR "two step"[Title/Abstract])) OR ((("component separation"[Title/Abstract]) OR "transversus abdominis"[Title/Abstract]) OR "retrorectus"[Title/Abstract])) OR (((("bridging"[Title/Abstract]) OR "bridge repair"[Title/Abstract]) OR "bridged repair"[Title/Abstract]) OR "silo"[Title/Abstract])) OR (("open"[Title/Abstract]) OR "laparoscopic"[Title/Abstract]))) AND (((((((hernia[Title/Abstract]) OR "abdominal wall defect"[Title/Abstract]) OR "abdominal wall reconstruction"[Title/Abstract]) OR "ventral defect"[Title/Abstract]) OR "enterocutaneous fistula"[Title/Abstract])) OR ("Hernia"[Mesh] OR "Hernia, Abdominal"[Mesh] OR "Hernia, Ventral"[Mesh] OR "Hernia, Umbilical"[Mesh]))

Filters: Publication date from 1995/01/01 to 2017/12/31; Humans; English; Adult: 19+ years
