# Supplementary material for: A systematic methodological review of non-randomised interventional studies of elective ventral hernia repair: clear definitions and a standardised minimum dataset are needed
Source: Hernia. 2019 May 31;23(5):859–72. doi: 10.1007/s10029-019-01979-9 (PMC6838456; doi:10.1007/s10029-019-01979-9)
Supplement: Supplementary file 2 — Supplementary material 2 (PDF 406 kb) [file 10029_2019_1979_MOESM2_ESM.pdf]

Online supplementary resource 2

A systematic methodological review of non-randomised interventional studies of elective ventral hernia repair: Clear definitions and standardised datasets are needed

Parker S.G[1], Halligan S[2], Erotocritou M[1], Wood C P J[1], Boulton R.W[1], Plumb A A O[2], Windsor A C J[1], Mallett S[3].

1. The Abdominal Wall Unit UCLH, GI Services Department, University College London Hospital, 235 Euston Road, London, NW1 2BU.
2. UCL Centre for Medical Imaging, 2nd Floor Charles Bell House, 43-45 Foley Street, W1W 7TS.
3. The Institute of Applied Health Research, College of Medical and Dental Sciences, University of Birmingham, Edgbaston, Birmingham, B15 2TT.

Corresponding Author: Mr Samuel G. Parker
Email: samgparker@nhs.net, Mobile: 07814136705 ORCID: 0000-0002-3710-9953

Data Extraction Sheet for Systematic Methodological Review of non-randomised Interventional Trials in Ventral Hernia repair.

Based on the ROBINS-I/ACROBAT assessment tool, Clinical reporting guidelines for cohort studies (STROBE), Template for intervention description (TIDieR), Downs and Black, Newcastle Ottawa, Expert opinion/knowledge of the literature.
Comparing 26 prospective interventional trials published since 2005, with 26 retrospective interventional trials (out of 94) published since 2005. Papers will be matched primarily according to Journal and the closest publication date, if no retrospective trial published in the same journal in the same year, then the prospective trial is matched with a trial performed in the similar impact journal, preferably published that year.

Reviewer details:

Reviewer:	
Date of data extraction:	

Study demographics

Multi or single centre	
Country of study	
Year of publication	
Number of Surgeons	
Consultant Surgeons or Trainees or both	
Paper number:	

Author of paper:	
Journal	
Impact factor	
Vol and page no	
Comparison groups	
Number of participants	
Ethical approval	

Inclusion Criteria:

Does the paper fulfil the following criteria:	Yes	No
▪ Published from 2005 onwards		
▪ Compares two different interventional techniques for ventral hernia repair		
▪ Aim of the study is to compare the outcomes of the two interventional techniques		
▪ Non-randomised participants		
▪ Published in English		

Introduction

Did the study report?	Yes	No
▪ A scientific rationale for the study?		
▪ A primary aim or objective?		
▪ A pre-specified hypothesis?		
▪ If a hypothesis was mentioned was there reference to the literature?		

Method

Study design

Did the study report?	Yes	No	Unclear	Page No.
▪ Was the data collected according to a protocol?				
▪ Description of equipment used? (Criteria see Appendix 1)				
▪ Detailed description of the interventions? (Criteria see Appendix 2)				
▪ Description of the primary outcome				
▪ Sample size/power calculation				

Participants

Did the study report?	Yes	No	Unclear	Page No.
▪ Is there any apparent selection other than ventral hernia, time, and place? (Yes if criteria are mentioned)				
▪ Reports a basic list of demographics? (Criteria see Appendix 3)				
▪ Were the baseline characteristics measured the same in both groups? (the ones measured)				
▪ Number of patients meeting the inclusion criteria (eligibility)				

▪ Number of patients included				
▪ Number of previous hernia repairs reported				
▪ Hernia maximal width reported				
▪ Hernia defect area reported				
▪ Mentions whether participants had primary VHs, incisional VHs or both?				
o If so which one?				
▪ Was a hernia grading scale used?				
o If so which one?				
▪ Participant recruitment - start date?				
▪ Participant recruitment - finish date?				
▪ Participant recruitment - end of follow-up date?				
▪ Where deviations from intended intervention reported?				

Reported outcomes

Did the study report?	Yes	No	Unclear	Page No.
▪ Was there blinding of the outpatient assessor to the intervention received?				
▪ Was there blinding of the participant to the intervention received?				
▪ Is hernia recurrence reported by intervention?				
▪ Was hernia recurrence defined?				
▪ Was a standardised definition of hernia recurrence used and referenced?				
▪ Was the length of follow up the same between groups?				
▪ Was a hernia detection method reported?				
o Questionnaire?				
o Telephone?				
o Clinical assessment only?				
o Clinical assessment +/- USS?				
o Clinical assessment +/- CT?				
o Re-operation rate?				
o Other?				
▪ What was the mean length of follow up?				
▪ Reoperation rate reported by intervention?				
▪ Surgical site infection reported by intervention?				
▪ Was surgical site infection defined?				
▪ Was a standardised definition of surgical site infection used and referenced?				
▪ Was a surgical site infection grading scale used?				
▪ Surgical site occurrence reported by intervention?				
▪ Was surgical site occurrence defined?				
▪ Was a standardised definition of surgical site occurrence used and referenced?				
▪ Were patient reported outcomes included?				
▪ If so which patient-reported-outcomes questionnaire was used?				
▪ Was a post-operative pain score used?				

Statistics

Does the paper fulfil the following criteria:	Yes	No	Unclear	Page No.
▪ Report the length of follow-up				
▪ Report the number of participants with missing data?				
▪ Report adjusted analysis				
▪ Report adjustment factors for adjusted analysis				
▪ Report estimate confidence intervals				
▪ Avoids restricting data analysis to patients with no missing data (complete case analysis)				
**Studies conducting prognostic analysis**				
▪ Report prediction estimates for standard clinical variables (Appendix 3)				
▪ Avoids all reported predictors having a statistically significant effect				

Appendix 1. – Description of the equipment used

Description of the equipment used contains details about:

1) The type of mesh used (material specified)
2) The type of suture used (if not the specific material - details whether absorbable or non-absorbable)
3) The type of tacks used (if not the specific material - details whether absorbable or non-absorbable)

-The authors define an adequate description of the intervention as containing at least 2 out of 3 of these items.

Appendix 2. – Description of the intervention/s performed

Description of the intervention/s contains details about:

1) Whether the operation was open or closed?
2) Whether the operation was with or without mesh?
3) Whether the operation was with or without a component separation?
4) The plane used for mesh insertion?
5) Whether the defect was closed or bridged?
6) Mesh fixation technique?
7) Concomitant procedures described such bowel resection or panniculectomy?
8) Drains used or not?

-The authors define an adequate description of the intervention as containing at least 4 out of 8 of these items.

Appendix 3. – Minimum list of participant demographics (any less than this list then the paper does not report enough informed)

Demographics table must contain the below three criteria:

Age
Sex
BMI

Demographics table must contain 2 out of the following 5 criteria:

Smoker
Diabetes
ASA
Primary hernia repair, or Incisional hernia repair, or both
Previous hernia repair
