# Supplementary material for: A systematic methodological review of non-randomised interventional studies of elective ventral hernia repair: clear definitions and a standardised minimum dataset are needed
Source: Hernia. 2019 May 31;23(5):859–72. doi: 10.1007/s10029-019-01979-9 (PMC6838456; doi:10.1007/s10029-019-01979-9)
Supplement: Supplementary file 3 — Supplementary material 3 (PDF 392 kb) [file 10029_2019_1979_MOESM3_ESM.pdf]

Online supplementary resource 3

A systematic methodological review of non-randomised interventional studies of elective ventral hernia repair: Clear definitions and standardised datasets are needed

Parker S.G[1], Halligan S[2], Erotocritou M[1], Wood C P J[1], Boulton R.W[1], Plumb A A O[2], Windsor A C J[1], Mallett S[3].

1. The Abdominal Wall Unit UCLH, GI Services Department, University College London Hospital, 235 Euston Road, London, NW1 2BU.
2. UCL Centre for Medical Imaging, 2nd Floor Charles Bell House, 43-45 Foley Street, W1W 7TS.
3. The Institute of Applied Health Research, College of Medical and Dental Sciences, University of Birmingham, Edgbaston, Birmingham, B15 2TT.

Corresponding Author: Mr Samuel G. Parker
Email: samgparker@nhs.net, Mobile: 07814136705 ORCID: 0000-0002-3710-9953

Methodological criteria: Introduction	No. of Prospective studies reporting criteria	No. of Retrospective studies reporting criteria	Total
Scientific rationale	25 (100%)	25 (100%)	50 (100%)
Primary aim or objective	18 (72%)	11 (44%)	29 (58%)
Pre-specified hypothesis	1 (4%)	2 (8%)	3 (6%)
Hypothesis referenced in the literature?	0 (0%)	0 (0%)	0 (0%)

Methodological criteria: Study design	No. of Prospective studies reporting criteria	No. of Retrospective studies reporting criteria	Total
Was a protocol written?	0 (0%)	0 (0%)	0 (0%)
An accurate description of the equipment?	17 (68%)	2 (8%)	19 (38%)
A detailed description of interventions	20 (80%)	16 (64%)	36 (72%)
Study has a primary outcome?	8 (32%)	10 (40%)	18 (36%)
Was a power calculation performed?	2 (8%)	0 (0%)	2 (4%)

Methodological criteria: Participants	No. of Prospective studies reporting criteria	No. of Retrospective studies reporting criteria	Total
Other criteria apart from elective ventral hernia repair?	18 (72%)	17 (68%)	35 (70%)
Reports a basic list of baseline characteristics?	7 (28%)	10 (40%)	17 (34%)

Were the baseline characteristics the same in both groups?	12 (48%)	6 (24%)	18 (36%)
Reported eligibility?	4 (16%)	2 (8%)	6 (12%)
Reported no. included?	25 (100%)	25 (100%)	50 (100%)
Reported no. previous hernia repairs?	6/20 (30%)	12/22 (55%)	18/42 (43%)
Reported maximal hernia width?	12 (48%)	8 (32%)	20 (40%)
Reported hernia defect area?	9 (36%)	12 (48%)	21 (42%)
Mentions whether primary or incisional or both are included?	18 (72%)	14 (56%)	32 (64%)
If so which type?			
Primary VH	1 (4%)	2 (8%)	3 (6%)
Primary umbilical	2 (8%)		2 (4%)
Primary incisional	2 (8%)	1 (4%)	3 (6%)
Incisional	6 (24%)	5 (20%)	11 (22%)
Both primary and incisional	7 (28%)	6 (24%)	13 (26%)
Hernia grade used?	2 (8%)	1 (4%)	3 (6%)
If so which hernia grade?			
EHS	1 (4%)	1 (4%)	2 (4%)
Adhoc (hernia widths)	1 (4%)		
Reported recruitment start date?	19 (76%)	17 (68%)	36 (72%)
Reported recruitment finish date?	19 (76%)	17 (68%)	36 (72%)
End of follow-up reported?	0 (0%)	0 (0%)	0 (0%)
Reported deviations from the intended intervention?	10 (40%)	8 (32%)	18 (36%)

**Methodological criteria: Reported Outcomes**	**No. of Prospective studies reporting criteria**	**No. of Retrospective studies reporting criteria**	**Total**
Blinding of the outpatient assessor?	3 (12%)	0 (0%)	3 (6%)
Blinding of the participant?	0 (0%)	0 (0%)	0 (0%)
Length of follow-up the same in both groups?	14 (56%)	15 (60%)	29 (58%)
Re-operation rate?	17 (68%)	13 (52%)	30 (60%)
**1)Hernia recurrence reported?**	25 (100%)	22 (88%)	47 (94%)
Hernia recurrence defined?	4 (16%)	5 (20%)	9 (18%)
Definition referenced?	0 (0%)	0 (0%)	0 (0%)

Was the hernia recurrence detection method reported?	18 (72%)	19 (76%)	37 (74%)
**2)Surgical site infection reported?**	16 (64%)	16 (64%)	32 (64%)
Surgical site infection defined?	3 (12%)	3 (12%)	6 (12%)
Definition referenced?	2 (8%)	1 (4%)	3 (6%)
Surgical site infection grade used?	1 (4%)	1 (4%)	2 (4%)
**3)Surgical site occurrence reported?**	1 (4%)	3 (12%)	4 (8%)
Surgical site occurrence defined?	0 (0%)	1 (4%)	1 (2%)
Definition referenced?	0 (0%)	0 (0%)	0 (0%)
Were patient reported outcomes included?	7 (28%)	3 (12%)	10 (20%)
EQ-5D?	2 (8%)		2 (4%)
Adhoc functional questions?	5 (20%)	2 (8%)	7 (14%)
French Hernia Club questionnaire?		1 (4%)	1 (2%)
Was a VAS score used?	6 (24%)	3 (12%)	9 (18%)

**Methodological criteria: Statistics**	**No. of Prospective studies reporting criteria**	**No. of Retrospective studies reporting criteria**	**Total**
Reported the length of follow-up?	23 (92%)	22 (88%)	45 (90%)
Reported the no. of participants with missing data?	8 (32%)	7 (28%)	15 (30%)
Reported adjusted analysis?	3 (12%)	7 (28%)	10 (20%)
Reported adjustment factors for adjusted analysis?	3 (12%)	5 (20%)	8 (16%)
Report estimate confidence interval?	2 (8%)	6 (24%)	8 (16%)
Avoids restricting analysis to patients with no missing data?	1 (4%)	0 (0%)	1 (2%)

**Methodological scores by criteria**	**Prospective studies (median, IQR)**	**Retrospective studies (median, IQR)**	**Total score**
Introduction	2 (1-2)	1 (1-2)	2 (1-2)
Study design	1 (1-2)	1 (1-2)	2 (1-3)
Participants	7 (6-8)	6 (5-8)	7 (6-8)
Reported outcomes	4 (3-5)	4 (3-6.5)	4 (3-6)
Statistics	1 (1-2)	2 (1-3)	1 (1-2)
Total with statistics score	17 (14-18)	13 (11-15.5)	15 (12-17.25)

Total	17 (14-18)	15 (12-18)	16 (14-18)
Total (mean, SD)	16.96 (4.01)	15.4 (3.45)	16.16 (3.79)

Hernia recurrence

**Prospective studies**	**Hernia recurrence definition**	**Definition referenced?**
Kurmann et al. (2)	'Recurrence was defined as any abdominal wall gap with or without bulge that is not covered by mesh in the area of the postoperative scar'.	No
Anadol et al	'Recurrence was defined as the presence of a defect and/or lump in the original location'.	No
Moreno-Egea et al	'Hernia recurrence was defined on physical examination and confirmed on CT'.	No
Boccicchio et al	'We defined a true hernia recurrence as herniation of bowel or omentum through a defect in the biological mesh or through a defect at the mesh/fascial interface after the initial operation'.	No
**Retrospective studies**	**Hernia recurrence definition**	**Definition referenced?**
Al-Salamah et al	'Recurrence was defined as any fascial defect, palable or detected on CT scan and located within 7cm of the site of hernia repair'.	No
Jin et al	'Patients with recurrent hernias were defined as requiring another hernia reoperation or noting a significant bulge'.	No
Ballem et al	'recurrence was defined by the presence of a new or similar bulge which increased in size upon straining'.	No
Booth et al	'Recurrent hernia was a contour abnormality associated with a fascial defect'.	No
Iacco et al	'Recurrence was defined by the presence of a bulge on physical examination, imaging, or by patient self-reporting'.	No

Surgical site infection

**Prospective studies**	**Surgical site infection definition**	**Definition referenced?**
Kurmann et al. (2)	'Surgical site infections were assessed according to the criteria developed by the Centers for Disease Control and Prevention (CDC)'.	Yes
Boccicchio et al	'Surgical site infection as defined by Centers for Disease Control criteria'	Yes
Winsnes et al	'defined as a wound infection treated with antibiotics'	No
**Retrospective studies**		
Al-Salamah et al	'Wound infection was defined as systemic features associated with tender swelling, with or without apparent discharge, necessitating open drainage'.	No

Ballem et al	‘Our definition of a wound infection was quite liberal and based on National Surgical Quality Improvement Program (NSQIP) recommendations for surgical site infections’.	Yes
Froylich et al	‘Patients with wound infections were considered positive if there was at least wound cellulitis, at which point antibiotic treatment was initiated’.	No

Surgical site occurrence

**Prospective Studies**	**Surgical site occurrence definition**	**Definition referenced?**
Nil	Nil	Nil
**Retrospective studies**		
Azoury et al	‘Wound complications included any surgical site occurrence post-operatively which delayed or hindered primary wound healing, such as abscess, seroma requiring drainage, dehiscence, necrosis, cellulitis, and hematoma’.	No

Hernia recurrence detection method

	**Prospective studies**	**Retrospective studies**	**Total**
Clinical assessment +/- CT	6	1	7
Clinical assessment +/- USS	5	1	6
Clinical assessment	3	-	3
Clinical assessment +/- telephone	1	3	4
Clinical assessment +/- USS/CT	1	1	2
Clinical assessment +/- USS/CT +/- clinical records	1	-	1
Re-operation rate	1	-	1
Telephone	-	1	1
Telephone + clinical records	-	2	2
Clinical records	-	3	3
Clinical assessment +/- CT +/- telephone	-	3	3
Clinical assessment +/- CT/USS +/- reoperation	-	1	1
Prospective database +/- clinical records	-	1	1
Prospective database +/- clinical records +/- CT	-	1	1
Prospective database +/- re-operation rate	-	1	1

**Mean length of follow-up**	Prospective studies	Retrospectives studies	Total
Recurrence <= 6months	1		1
Recurrence >6months,≦12months	4	6	10
Recurrence >12months,≦18months	3	2	5
Recurrence >18months,≦24months	4	2	6
Recurrence >24months,≦36months	6	3	9
Recurrence >36months,≦48months	2	1	3
Recurrence >48months, ≦60months	1	4	5
Recurrence >60months	3	3	6
Unclear	2	3	5
